# Biography of Dr. Maton

**Published:** 1835-07-01

**Authors:** 


### BIOGRAPHY OF DR. MATON.

(FROM A CORRESPONDENT.)

THE wish expressed by the Editor of the Medical Quarterly Review, that some record of the late Dr. Maton should be entered on its pages, is in itself an acknowledgment of the useful influence which this good man exercised on our common profession. The life of Dr. Maton was not eventful, and his personal character, which formed its chief distinction, could in no way be inferred by the detail of its incidents. To that character I shall address my remarks. The esteem in which it was held by his brethren of the profession received its most eloquent illustration, in the sorrow which prevailed among those assembled at the College of Physicians, on the evening of the day on which he died. The expressions of regret for his loss were universal, and the feeling which prompted them was, in most instances, evidently deep and sincere. His life had been a reproof to many. By the good and the wise, by the earnest and the humane, by those like himself, it was then said, "We have lost from our society the one whom, taking him all in all, we could perhaps at this time the least spare. A physician, who in kindness and honour always did his best for his patient,—a brother whom we could trust,—a man on whose word we could depend." Alas! that such qualities as benevolence and integrity of purpose, assumed, in vulgar phrase, to be in all places common, and frequent as every day's life,—alas! that they should still in truth be rare, and confer distinction in our chronicle of the dead. When we mourn for one like him of whom I write, there is consternation in our sorrow. Why was Dr. Maton's life thus honoured in its close by the opinions of those with whom he had lived? How had his good name been secured? The answer is ready with all who knew him —in respecting himself he respected others. It was not by mere cleverness, by real or affected brilliancy of talent, that he won favour from the professional mind,—by no specific attribute of character or manner addressed to their vanities and infirmities,—but rather by the application of a wholesome example; by an influence constant as that of the air, mild and insensibly alterative as that of the best medicines when best administered.

Dr. Maton was popular in the profession, but his popularity was not of the common, spurious kind. It was the result of good manners and of fair dealing, of integrity and firmness of principle. It was not coaxed out of others by indiscriminate familiarity, or purchased from them by an overlavish hospitality. He did not toil for universal favour by the laborious selfishness of universal good-nature, nor did he advertise for flattery by the habit of complimenting himself. The favour for which so many hunt abroad, pursued him to his retirement.

In his professional relations with the public, perhaps no physician ever acquired more individual confidence, or secured more attachment. His attention to the complaints of his suffering clients was seen to be close, his sympathy was felt to be real. In his intercourse with the sick, he pursued no unworthy arts, and sought favour by no indirect means; ever mindful of their claims on his humanity, and of the dignity of his own position. His was not the poor vanity of buying from the affluent sick a spurious character for generosity, by refusing at undue seasons the compensation which was his due, to the detriment of those who acted with him. Of necessity, under the general laws of society, and of our profession as a section of it, being good and eminent he was disparaged by exceptions, and these his own good qualities were made to furnish. His moral excellence being acknowledged, his physic was therefore questioned into mediocrity. It has been said of him, in flippant detraction, that he was "a good man, but no physician;" "truly a most worthy gentleman, but deficient in medical science." That he was considered a good, and an eminently good physician, by the London public, is known to us all; but surely we may assume, that being a man of good abilities and sound judgment, of benevolence and industry, with large opportunities, practising honestly and earnestly, he thus, *de facto*, became of necessity a good physician. Undoubtedly he was a better physician than some of those who disparaged him. His respect for himself, and for the art which he practised, would not suffer him to substitute assertion for argument, or vague surmises for deliberate opinion, in the consultations at which he assisted; thus he was often silent, when the brawler was heard.

Dr. Maton was conservative, rather than reforming, in his general tendencies, but he did not, therefore, refuse all change; and his reform, as far as it went, could always be trusted. Three years back, before dissection was legalised under Mr. Warburton's bill, when murder and sacrilege ministered to science, and the anatomist waited on the public executioner, Dr. Maton was forward in applying by earnest petition to the Legislature, for the protection of his medical brethren against the wrongs and outrages by which they were assailed. Among the older physicians of London, Dr. Maton was, in truth, distinguished by liberality of sentiment, rather than by the opposite qualities of mind.

In all things Dr. Maton was good and kind. The love of Nature led him annually to her streams and mountain haunts, in this island and abroad. The study of flowers he felt to be an amiable pursuit, and therefore he became a botanist. His courtesy was of the old school, sincere, and ever watchful in reciprocating that of others. In the busiest season of the year, every note addressed to him was answered, every visit was returned. When his time became most valuable, Dr. Maton could yet always find leisure to acknowledge the most trifling civility. His extreme punctuality in appointments will long be remembered by the profession. It was remarkable, to the extent of peculiarity, but, like his love of order and neatness, it had its source in a regard for the convenience of others, and became him in his double capacity of physician and bachelor. Yet, because Dr. Maton's character was essentially amiable, let us not suppose that it was therefore of the negative kind: its tone was firm and masculine, in harmony with that of Baillie and Heberden, of whom he was the chosen friend and associate.

By the young let it be remembered, in his epitaph, that he watched with loyal affection over the infancy and girlhood of their future Queen; while those of mature judgment, who knew him not, will find their best authority for his virtues and good sense in the confidence with which he was honoured by her illustrious mother.

I had not intended to have remarked on any particular indication of his character, but to one let me briefly allude. Dr. Maton, early in life, made himself responsible for large debts which his father had incurred, and subsequently discharged them, with their accumulated interest. But I pause in the reproof, that I am dwelling on the recollection of virtues which have never been questioned; that I am but repeating the praises which others have always been eager to concede to the excellence of my departed friend.

I find, on reviewing my portrait, that I have traced no lineament in his character, with which all who knew him have not long been familiar; that I have ascribed to him no qualities which were not his by acclamation. It was one of his distinctions that, while living, he was honoured by those with whom he lived.

Let us confidently presume, that in the annals of the College over which he occasionally presided, his virtues found an early record, and that at the " Comitia" first held after his decease, the name of Dr. Maton was mentioned by his associates for honour and in sorrow.

" Næ illiusmodi jam magna nobis civium
Penuria est—homo antiquâ virtute ac fide!"

*May Fair; June*, 1835.

